# Bilateral Scapular Fractures Occurring as a Result of a First-Time Seizure

**DOI:** 10.1155/2022/9186275

**Published:** 2022-05-17

**Authors:** David P. Betten, Ian S. Batson, Leah N. Babiarz, Kristen N. Owen

**Affiliations:** ^1^Sparrow Health System, Department of Emergency Medicine, 1215 Michigan Ave, Lansing, Michigan 48912, USA; ^2^Michigan State University College of Human Medicine, Department of Emergency Medicine, 965 Wilson Road Suite A110, East Fee Hall, East Lansing, MI 48824, USA

## Abstract

The violent nature of generalized tonic-clonic seizures puts individuals at risk of a large number of potential injuries. These can occur due both to the profound muscular contractions that accompany these episodes as well as falls and other traumatic events that occur due to the period of loss of consciousness that occurs during generalized seizures. While injuries such as soft tissue contusions, tongue biting, dental injuries, and facial lacerations resulting from falls from standing predominate, bony injuries are not uncommon. We present a case of bilateral scapular fractures that occurred in an otherwise healthy 32-year male who presented with shoulder and back pain and inability to perform any significant movement of his upper arms secondary to pain after experiencing an apparent first-time generalized tonic-clonic seizure. The presence of unilateral and bilateral scapular fractures, while uncommonly described, should be considered as an additional potential orthopedic injury that may occur secondary to a generalized tonic-clonic seizure. In the absence of observed significant forceful traumatic injury, this injury is unusual, and its presence noted in a patient experiencing sudden loss of consciousness should raise heightened concern of seizures as the potential etiology.

## 1. Introduction

There exists the potential for injury accompanying generalized tonic-clonic seizure activity due to both the strong muscular contractions that define these episodes in addition to the lack of natural protective behaviors one might typically rely upon to lessen injuries occurring from falls during this period of loss of consciousness. Loss of shoulder mobility following a generalized tonic-clonic seizure should lead clinicians to evaluate for the potential for anterior as well as the classically described posterior shoulder dislocation. In the absence of a glenohumeral dislocation, other injuries involving the shoulder girdle, including scapular fracture, should be considered. We present a case of bilateral scapular fractures in an otherwise healthy young male following a new onset generalized seizure.

## 2. Case Presentation

A 32-year-old male with a history of depression, taking solely bupropion, presented to the Emergency Department (ED) due to an episode of loss of consciousness and inability to lift his arms. He was communicating via computer with others when he sensed a brief uneasy feeling followed by abrupt loss of consciousness. Individuals conversing with him online described retching sounds, fast and irregular breathing, and then absent patient communication for approximately thirty minutes. After this time, he was described to have slurred and nonsensical speech followed by a gradual return to his baseline mental status. Upon regaining consciousness, the patient noted he was on the floor next to his ground level chair. While able to use his hands to operate his phone, he had otherwise limited range of motion and significant pain and limited range of motion of his upper extremities.

**Figure 1:**
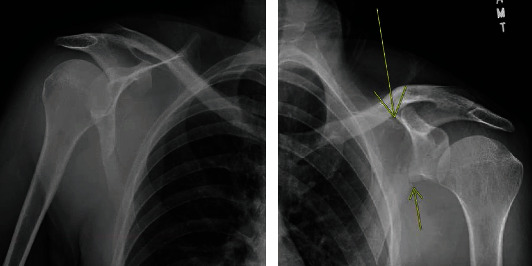
Plain film radiographs of the right shoulder with no obvious right bony abnormalities noted. Left-sided scapular body fracture identified with intact glenohumeral joint.

**Figure 2:**
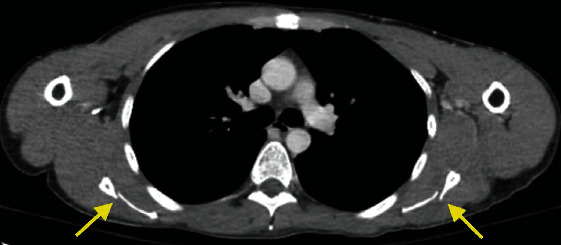
Computed tomography (CT) imaging demonstrating bilateral scapular body fractures.

**Figure 3:**
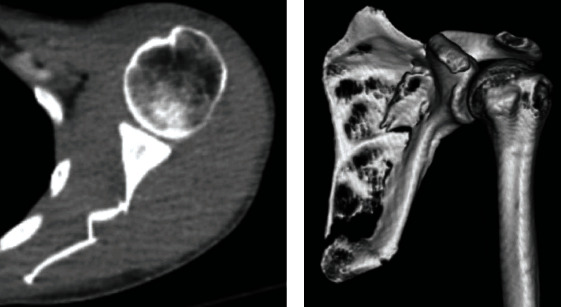
Standard CT and CT reconstruction image demonstrating more extensive left scapular body fracture.

Upon ED presentation, he demonstrated a clear sensorium and complained solely of severe bilateral shoulder and upper back pain. Vital signs included a temperature of 98.9 Fahrenheit, blood pressure of 160/89 mmHg, pulse of 107 beats per minute, and a pulse oximetry of 96%. There was no external evidence of trauma aside from a small contusion under his left eye. Shoulder range of motion was limited due to pain. Upper extremity sensation, peripheral pulses, and forearm and hand strength were normal. Laboratory studies including a urine drug screen were unremarkable. Plain radiographs of the shoulders showed an intact glenohumeral joint with a suspected left-sided scapular body fracture (Figure 1). A subsequent CT of the chest demonstrated bilateral scapular fractures (Figures 2 and 3). Neurology consultation was obtained with an impression of a generalized seizure having occurred given the historical components of the patient's presentation and injury pattern. An MRI brain and EEG performed were unremarkable. The patient was gradually discontinued from his bupropion and placed on an upward taper of lamotrigine while temporarily being placed on levetiracetam. Bilateral scapular fractures were managed nonoperatively with a return to his baseline over 6 weeks.

## 3. Discussion

Traumatic injuries in patients with epilepsy are not uncommon, occurring in 23% of individuals within the first 2 years of their diagnosis [1]. Longitudinal studies note a long term increased predilection for drownings, motor vehicle accidents, and burns, as well bony and soft tissue injuries. While behavior modification may reduce this risk, the potential for soft tissue and bony injuries is difficult to fully eliminate [2]. The observed increased risk of facial and dental injuries, lacerations, and distal extremity fractures can be surmised to occur related to loss of consciousness and absence of reflexive protective measures that minimize trauma occurring during falls.

The significant musculature contraction occurring during the tonic phases of seizures is of greatest force in the axial and proximal extremity muscle groups that may lead to fractures that occur unrelated to those occurring from direct impact trauma. These common injuries include anterior and posterior shoulder dislocations and fractures, thoracic and lumbar compression fractures, and proximal femur and acetabular fractures [3]. While posterior shoulder dislocations, with or without fractures, are commonly reported, their occurrence remains low noted in less than 0.6-0.9% of patients with seizures, most commonly occurring in the setting of a patient's first seizure [4, 5]. Associated fractures are common, including the reverse Hills-Sachs and reverse Bankart lesions, which occur in up to 65% of posterior dislocations [6].

While seizures are the etiology for nearly one-third of posterior shoulder dislocations, unilateral and bilateral scapular fractures are infrequently reported outside of the setting of high energy trauma. A review of 17 patients with bilateral scapular fractures described 6 patients without associated high impact trauma of which 2 were due to seizure and 4 were secondary to electrical shock [7]. Additional cases of bilateral scapular fracture identified include three individuals who were hemodialysis dependent individuals with renal osteodystrophy [8–10]. Associated risk factors for all fracture types in patients with epilepsy, in addition to renal osteodystrophy, include older age and osteoporosis and hyperparathyroidism, as well as anticonvulsant therapy which may be lead to reduced bone density [8, 11, 12]. Our case is just the second identified in the literature in which bilateral scapular fractures occurred in a relatively young and healthy individual who was interestingly also participating in computer-based activities at the time of seizure onset [13].

Plain radiographs may be considered initially for those patients in which glenohumeral dislocation is considered the most likely diagnosis. However, identifying scapular fractures on plain film radiography may be challenging, missing up to 43% of fractures in patients suffering from traumatic injuries [14]. Seizures have been identified as associated with a delay in diagnosis compared to those patients who experienced typical traumatic mechanisms [15]. Dedicated scapula view radiographs or preferably CT scans will identify potentially occult scapula fractures and should be strongly considered as an additional possible etiology for shoulder and upper back pain following seizure activity [16].

Scapular fractures should be considered as a potential etiology for shoulder and upper back pain following seizure activity. Additionally, the presence of this injury following loss of consciousness in the absence of significant trauma should prompt the clinician to consider seizures as a precipitant event.

## Data Availability

No data was used to support this study.
